# Feeding Ractopamine Improves the Growth Performance and Carcass Characteristics of the Lard-Type Mangalica Pig

**DOI:** 10.3390/ani13243857

**Published:** 2023-12-15

**Authors:** Maegan A. Reeves Pitts, Hunter R. Smith, Ellie C. Amerson, Jessica D. Starkey, Charles W. Starkey, Jason T. Sawyer, Terry D. Brandebourg

**Affiliations:** 1 Department of Animal Sciences, Auburn University, Auburn, AL 36849, USA; 2 Department of Poultry Science, Auburn University, Auburn, AL 36849, USA; jessica.starkey@auburn.edu (J.D.S.);

**Keywords:** Mangalica, swine, ractopamine, growth performance, pork quality

## Abstract

**Simple Summary:**

A resurgence in U.S. niche markets catering to customers demanding high-quality pork products has renewed U.S. producers’ interest in lard-type, heritage breeds such as the Mangalica pig. However, the excessive amount of lard associated with Mangalica production poses a limitation to the adoption of this breed. This study aimed to determine if feeding 20 mg per kg of ractopamine hydrochloride (RAC) to growing Mangalica pigs would improve their growth performance without impairing carcass merit. Feeding RAC improved both Average Daily Gain and gain efficiency while increasing loin eye area and promoting darker objective color scores. These changes occurred without impacting marbling score or measures of tenderness. Therefore, feeding RAC may be a viable strategy to improve the economic feasibility of utilizing this breed to target niche markets.

**Abstract:**

Mangalica pigs are gaining popularity within the U.S. as a niche breed, given their reputation for superior-quality pork. However, slow growth rates, a poor lean yield, and excessive adiposity limit the widespread adoption of Mangalica. To determine if feeding the metabolic modifier, ractopamine hydrochloride (RAC), would improve growth performance without impairing pork quality in the Mangalica, pigs were fed either 0 or 20 mg per kg RAC for 21 days. At 24 h postharvest, pork quality and carcass composition measurements were recorded; then, primal cuts were fabricated and assessed. RAC increased ADG (*p* < 0.04) and gain efficiency (*p* < 0.03) by 24% and 21%, respectively. RAC increased Loin Eye Area (*p* < 0.0001) by 21% but did not impact the 10th rib fat depth (*p* > 0.90) or marbling score (*p* > 0.77). RAC failed to alter any primal cut weights. Feeding RAC lowered b* values (*p* < 0.04) and tended to lower L* values (*p* < 0.08) while not affecting a* values (*p* > 0.30), suggesting RAC darkened loin color. Finally, RAC decreased cook yield percentage (*p* < 0.02) by 11% without impacting Warner-Bratzler Shear Force (*p* > 0.31). These data support the hypothesis that feeding RAC to Mangalica improves growth performance without impairing pork quality in this breed.

## 1. Introduction

At the point of sale, consumer choice is heavily influenced by color attributes and the degree of marbling displayed by pork cuts [1]. However, selection for leaner, more rapidly growing animals by the pork industry has in some cases led to consumer perceptions of lower-quality products compared to other animal protein sources [2,3]. In response, niche markets have developed, whereby producers are rewarded for providing high-quality pork products that command premium prices [4]. These markets allow for small- and medium-sized producers to access these consumers via direct marketing to high-end restaurants, through online sales, or via local distribution, utilizing venues such as farmers’ markets or “locally grown” retailers [4]. Within the U.S., this trend has renewed the interest of producers in the adoption of heritage, lard-type swine breeds to meet these new market opportunities.

The Mangalica is well positioned as a niche breed, given it is a lard-type hog known for its high-quality lard that contributes to highly marbled, uniquely flavored meat cuts [5,6,7,8,9,10,11,12,13,14,15]. Recent research characterizing their growth performance and carcass merit confirms that Mangalica pork is more highly marbled and redder in color compared to modern breeds harvested at similar weights [16,17]. However, the superior quality associated with the higher propensity of Mangalica pigs to fatten compared to modern production breeds occurs at the expense of poorer growth performance, lower carcass cutability, and excessive accretion of trim or waste fat [5,6,7,8,9,10]. This poorer growth performance is also accompanied by delayed onset of puberty and smaller litter sizes, resulting in fewer offspring produced compared to modern production breeds [11,12,13,14,15]. These former attributes pose limitations to the adoption of this breed, given that, often, producers targeting niche markets do not have access to viable outlets that would allow them to profitably market the amount of lard associated with Mangalica carcasses.

Strategies that improve lean growth without impairing carcass merit would aid producers who utilize heritage breeds. β-adrenergic agonists (βAAs) are a class of compounds that, when fed to growing food animals, have been shown to orchestrate the repartitioning of nutrients away from fat deposition toward support of lean growth, ultimately leading to improvements in feed conversion [18,19]. As such, βAAs have been developed as feed additives meant to improve growth performance, and ractopamine hydrocholoride (RAC) is one such βAA that has been approved for use in swine [20]. Feeding RAC to finishing swine results in increased growth rate and heavier-muscled, leaner carcasses compared to their untreated counterparts while a meta-analysis of the RAC response indicated these positive changes in carcass merit were not associated with reduced marbling [18,19,21].

There is reason to believe that feeding RAC might not impair pork quality. Multiple studies have failed to observe an impact of RAC on postmortem muscle pH or measures of tenderness [22,23,24,25,26]. Importantly, a meta-analysis of the RAC response indicates feeding RAC appears to have no negative effects on either color attributes or marbling scores [21]. Such responses in finishing hogs suggest that using RAC as a feed additive in the diet of Mangalica pigs might be a strategy to help mitigate the challenges facing producers who are adopting this breed within the U.S.

Unfortunately, little information exists in the literature concerning the impact of growth promotants such as RAC on growth performance, body composition, and pork quality traits in the Mangalica pig. Our recent work indicates that, concerning carcass merit, there is little justification for harvesting Mangalica pigs at body weights greater than 80–100 kg because of the excessive fat accretion that occurs relative to the limited increase in leanness observed at heavier weights in this breed [17]. To assess the potential that feeding RAC is a viable strategy to limit excessive fat accretion without harming pork quality in heritage breeds, the RAC response needs to be better characterized in finishing Mangalica. Thus, our objective was to conduct a growth trial to determine if feeding RAC would improve growth performance without impairing pork quality in the Mangalica at weights below 100 kg. Growth performance, carcass composition, and meat quality parameters were then compared in RAC-fed animals versus their control-fed counterparts.

## 2. Materials and Methods

### 2.1. Animals and Design

All experimental procedures were approved by the Auburn University Institutional Animal Care and Use Committee. The Auburn University College of Agriculture is accredited by the Association for Assessment and Accreditation of Laboratory Animal Care International (AALAC), and this study was conducted in accordance with the Federation of Animal Science Societies’ Guide for the Care and Use of Agricultural Animals in Research and Teaching. A total of twenty-eight growing Mangalica pigs were obtained from the Auburn University research herd housed at the Auburn University Swine Research and Education Center. Pigs were individually housed in pens 12.2 m^2^ in size and were provided *ad libitum* access to food and water. Pigs with an initial body weight of 73.3 kg were randomly assigned to a typical finisher ration (14.8% crude protein, 4.6% fat, 3.47 mcal/kg metabolizable energy, 0.68% calcium, 0.45% available phosphorus) supplemented with either 0 or 20 mg per kg ractopamine hydrochloride (RAC; Elanco Animal Health, Greenfield, IN; n = 14 per treatment) for 21 days with gender evenly distributed across treatments (Table 1). The slow growth rates and low percentage of lean growth exhibited by this breed do not warrant supplementation of amino acids and phosphorus to the basal diet in the RAC-fed animals [6,16,17]. Daily feed intakes and weekly body weights were recorded for all animals in the test to facilitate measurement of average daily gain (ADG), gain efficiency (kg gained:kg feed), dressing percentage ((hot carcass weight/live weight) × 100)), and total feed intake. All pigs were then finished to a harvest weight of 105 kg.

### 2.2. Carcass Fabrication during Postharvest

All pigs were transported to the Auburn University Lambert-Powell Meats Lab where they were harvested following electrical stunning according to standards outlined by the USDA-FSIS inspection service. Hot carcass weight was recorded at the conclusion of harvest activities and prior to carcass washing/rinsing. Cold carcass weights were determined following carcass chilling at 2 °C for 24 h. Cold side weight and carcass length were determined at 24 h postmortem, following vertical splitting of carcasses. Primal cuts consisted of the ham (IMPS# 401), loin (IMPS# 410), shoulder (IMPS# 403), and belly (IMPS# 408). Institutional meat packer’s specifications (IMPS) for pork carcasses were followed to facilitate the individual cut fabrication and weight. Leftover trim and fat were likewise weighed for each individual cut after fabrication. Each primal weight was recorded using an analytical balance (PB3002-S, Mettler Toledo, Columbus, OH, USA) prior to packaging.

### 2.3. Carcass Composition Determination

From each loin, chops 2.54 cm thick (n = 8) were cut, individually identified, vacuum-packaged, and subjected to laboratory analysis of surface color, shear force, and cook loss, including proximate analysis. Fat back was resected from the loin before being weighed. Measures of fat depth were recorded at the 1st, 10th, and last ribs, including last lumbar [27,28]. Samples for proximate analysis (protein, moisture, fat, and collagen) were measured using an approved near-infrared (NIR) spectrophotometer (FoodScan™, FOSS Analytical A/S, Hillerod, Denmark), with data processing conducted by ISIscan™ Software version 6.0. These values represent relative estimates based on NIR values. Prior to analysis, each chop was ground using a stainless-steel Waring blender [29].

### 2.4. Carcass Merit Determination

To measure back fat (BF) depth along with loin eye area (LEA) according to NPPC guidelines for the estimation of quality, carcasses were split and separated between the 10th and 11th rib interface [30]. A trained observer using published visual standards determined subjective scores for marbling [28,29]. Hunter L*, a*, and b* values were measured using a Hunter Miniscan XE Plus (Hunter Lab, Reston, VA, USA) to evaluate objective color attributes of the longissimus muscle at the 10th rib [31]. A D65 light source, a 10° viewing angle, and a 35 mm viewing area were used to calibrate the Miniscan according to the manufacturer’s recommendations. At 24 h postmortem, a temperature-compensating pH Spear probe (Oakton Instruments, Vernon Hills, IL, USA) recorded the carcass ultimate pH in the loin muscle.

Warner Bratzler shear force (WBSF) evaluation was performed using pork loin chops cut to 2.54 cm thickness and thawed in a vacuum package bag at approximately 4 °C for 24 h. Chops were weighed on an analytical balance (PB3002-S, Mettler Toledo, Columbus, OH, USA) after removal from individual packaging and blotted drying. After weighing, each chop was placed onto a cooking rack and placed into a convection oven pre-heated to 177 °C. A copper constantan thermocouple wire was inserted into the geometric center of the chop and attached to a hand-held Omega data logger HH309A (Omega, Stamford, CT, USA) temperature recorder in order to monitor the temperature of each chop during cooking to ensure an internal temperature of 71 °C was achieved. Chops were then removed from the oven, allowed to cool to room temperature, and re-weighed. From each chop, six 1.27 cm diameter cores were removed with a brass cork borer (Model 1601A Series Brass Cork Borer, Boekel Scientific, Feasterville, PA, USA) parallel to the longitudinal orientation of the muscle fibers. Each core was sheared once at its center using a TA-XT2i Texture Analyser (Texture Technologies Corp., Scarsdale, NY, USA) with a 30 kg load cell. The peak force measurements were averaged from the six cores of each sample and were used for analysis. The penetration speed was 3.3 mm per second with a post-test speed of 10 mm/s and a pre-test speed of 2.0 mm per second. Cook yield was measured as the percentage of pre-cooked weight lost during cooking and calculated as Cook Yield = ((Raw weight − Cooked weight)/Raw weight)) × 100.

### 2.5. Statistical Analysis of Data

Data were analyzed using a completely randomized block design using a mixed linear model of SAS v9.2, with individual animals serving as the experimental unit, i.e., individual block (SAS Institute, Inc., Cary, NC, USA). Growth performance, carcass parameters, fabricated primal cut measurements, and 24 h post-harvest pH and color scores were analyzed as response variables. Statistical differences were declared when *p* ≤ 0.05.

## 3. Results

### 3.1. Growth Performance

The effect of RAC on growth performance in Mangalica pigs was determined by evaluating body weight, daily feed intake, ADG, and gain efficiency for animals during the 21-day feeding period (Table 2). Neither initial nor final body weights differed between groups. Likewise, daily feed intake was not different between control or RAC-fed animals (*p* > 0.71). However, ADG was increased by 24% (*p* < 0.0025) and FE was increased by 21% (*p* < 0.0029) in pigs fed RAC versus those on the control diet.

### 3.2. Carcass Parameters and Primal Cut Measurements

Hot carcass weight, cold carcass weight, carcass length, and the weight of primal cuts (ham, loin, shoulder, belly) were evaluated for control and RAC-fed pigs post harvest (Table 3). Neither hot carcass weight (*p* < 0.22), cold carcass weight (*p* < 0.25), nor carcass length (*p* < 0.26) were altered by RAC treatment. In the present study, RAC treatment did not alter primal cuts as the ham (*p* < 0.21), loin (*p* < 0.43), shoulder (*p* < 0.45), or belly (*p* < 0.73) weights were not different versus control pigs.

### 3.3. Carcass Composition

To determine the RAC response based on carcass composition in Mangalica, fat back (subcutaneous fat between the skin and longissimus dorsi muscle), fat depth along the vertebrate, average back fat, marbling score, muscle score, loin eye area, and nutrient content (collagen, fat, protein, and moisture) were evaluated for control and RAC-fed pigs post harvest (Table 4) according to established National Pork Board procedures [30]. Feeding RAC increased LEA by 21% (*p* < 0.0001) while muscle score was not changed (*p* < 0.22). Neither average back fat (*p* < 0.41) nor fat depth at the 1st rib (*p* < 0.31), 10th rib (*p* < 0.90), last rib (*p* < 0.60), nor last lumbar (*p* < 0.61) were altered by RAC. Importantly, marbling score was not altered by RAC (*p* < 0.77). Likewise, no differences were observed in collagen (*p* < 0.15), fat (*p* < 0.34), protein (*p* < 0.21), or moisture (*p* < 0.68) content in control versus RAC-fed Mangalica pigs.

### 3.4. Longissimus Dorsi (Loin Eye) Color and Ultimate pH (24 h)

Loin ultimate pH, objective color (L*, a*, b*), cook yield, and Warner–Bratzler Shear force (WBSF) were evaluated for control and RAC-fed pigs post harvest (Table 5). The 24 h pH was not significantly different across treatments (*p* < 0.47). On the other hand, the objective color measures L* (lightness) tended to decrease (*p* < 0.08), and b* (yellowness) significantly decreased in RAC-treated pigs (*p* < 0.04); however, a* (redness) did not change in response to RAC (*p* < 0.30). Finally, RAC decreased cook yield percentage (*p* < 0.02) by 11% while not impacting WBSF (*p* > 0.31).

## 4. Discussion

Few studies characterizing the growth performance of Mangalica pigs exist in the literature, with most focusing on their performance when reared in pasture-based production systems such as those typical of rural eastern European communities. We have recently conducted growth trials using Mangalica pigs fed a concentrated ration while reared in confinement within a closed herd as is typical of modern U.S. production facilities [16,17]. Under such conditions, the growth performance of Mangalica pigs is characterized by an ADG of 0.450 kg per day, a feed intake of 2.2 kg per day, and a gain efficiency of 0.230 [16,17]. In the current study utilizing similar conditions, pigs within the control group exhibited an ADG of 0.525 kg/day, daily feed intake of 2.23 kg per day, and an FE of 0.241. While these values are consistent with our previous studies, they slightly exceed the previous performance standards. This is likely due to the study design, as the current study assessed growth during the growing phase, a period during the pig’s developmental trajectory when growth rate is maximal. Our previous studies characterized growth across the growing and finishing stages, and it is likely that the slower growth associated with finishing contributed to the slightly lower performance in those studies [16,17]. Nonetheless, the growth performance of Mangalica in the current study was consistent with expected values suggesting that the RAC response observed in this study was not confounded by an abnormal growth response during the trial.

Ractopamine HCL (RAC) is a beta-adrenergic agonist that has been approved for use in U.S. swine diets as a growth promotant [20]. When fed to modern lean genetic pig breeds, RAC consistently improves feed efficiency by 10–20% across inclusion rates ranging between 5 and 20 mg per kg, with only a few studies reporting that RAC failed to improve this parameter [18,19,21]. This improvement is associated with a change in the composition of gain reflected by increased protein deposition and less lipid deposition, as indicated by an overall 5–24% increase in loin eye area and 5% increase in carcass dressing percentage, while concomitantly, feed intake is modestly reduced by 5–10% by RAC administration [18,19,21]. Generally, RAC increases ADG by 5–15% in pigs, but the response often fails to show dose dependence over increasing dietary inclusion rates within a study [24,25,26,32,33,34]. In the present study, RAC increased FE by 21% and ADG by 24% versus the control diet, though RAC did not affect feed intake. These results are consistent with the swine literature and indicate that the Mangalica are responsive to RAC as the increase in both FE and ADG is comparatively higher than those reported in the literature. The lack of effect on feed intake was somewhat surprising; however, the suppressive effect of RAC on feed intake in swine is generally mild [18,21]. Furthermore, some studies in the literature similarly failed to observe an effect of RAC on feed intake [33,34,35]. Interestingly, one study examining the RAC response in obese pigs reported a 6% decrease in response to RAC feeding compared to control animals [36]. The robust responses in other growth parameters in the present study indicate that RAC fed in the present study was clearly efficacious. Given that RAC suppressed feed intake in obese pigs, the inability of RAC to suppress feed intake in Mangalica in the present may be due to intrinsic biological factors of this breed, such as the genetic propensity of Mangalica to fatten or the potential that neural networks regulating voluntary intake in Mangalica differ from those in breeds that have undergone intensive selection for rapid growth. Nonetheless, these data indicate that feeding RAC to Mangalica is associated with improvements in growth performance and supports our hypothesis that RAC is a viable strategy to improve the profitability of Mangalica production.

Carcass cutability is another important trait that influences carcass value. Stites et al. [32] examined the impact of RAC on carcass cutting yields and observed an increase in trimmed hams and loins but no effect on the shoulder or belly of RAC-treated carcasses when feeding RAC to crossbred, meat-type hogs for 48 days, though the increase measured in the ham and loin cuts was modest. In the present study, RAC did not impact the weights of any primal cuts. Given that the Mangalica is a lard-type hog and RAC did not impact any measure of carcass fat, it is possible that the impact of RAC on the lean portion of the primal cut may have been obscured by the contribution of adipose tissue in each cut.

The impact of RAC on carcass merit is also an important consideration. Feeding RAC at a 10–20 mg per kg inclusion rate generally increases carcass merit through an increase in carcass leanness and carcass dressing percentage [18,19,20]. Interestingly, the impact of RAC on fat deposition is equivocal as RAC has been shown to decrease back fat depth by 10–15% in some studies, though several other studies failed to observe a RAC response on either backfat depth or the 10th rib fat depth [18,21,37,38,39,40,41,42,43]. In the current study, RAC did not impact any measure of carcass fat examined. Importantly, regardless of whether backfat depth was decreased or not, a meta-analysis of the RAC response in swine indicated that feeding RAC does not appear to impair marbling score [21]. In the present study, RAC increased LEA by 21% without altering marbling score. These results suggest a very robust muscle response to RAC in Mangalica pigs that does not impair the high levels of intramuscular fat displayed by this breed. The explanation for why RAC impacts cover fat in some studies and not others is not clear. The Mangalica has a high genetic propensity to fatten and, thus, its adipose tissue may not be as responsive to RAC as other leaner breeds appear to be based on the literature. Interestingly, despite an apparent lack of responsiveness in the adipose tissue, RAC significantly increased loin eye area in Mangalica. Overall, these data are consistent with published literature across breeds and indicate that feeding RAC to Mangalica improves carcass merit while the Mangalica exhibits a unique tissue-specific response to RAC.

Pork color is a key variable influencing consumer perception of pork quality [1,2]. In previous studies, we observed that Mangalica pork is a more desirable, darker-red color compared to modern meat breeds, suggesting a higher-quality pork product [16,17]. In the present study, objective color scores in control animals were consistent with our previous observations. Feeding RAC decreased both the objective color parameters L* and b* versus the control animals, indicative of promoting a darker color and that the RAC may further enhance this attribute in Mangalica pigs. These results broadly agree with the impact of RAC on objective color scores in the literature as well, whereby feeding RAC generally promotes darker objective color scores [21]. Multiple studies in the literature have also indicated that RAC does not influence objective measures of tenderness, water-holding capacity, or sensory panel scores of juiciness or tenderness [21]. Interestingly, in the current study, cook yield percentage was slightly lower in chops from RAC-treated pigs although WBSF, a measure of tenderness, was unaffected. Since sensory panel analysis was not conducted in the current study, it is not possible to know if the lower cook yield in RAC treated chops impacted perceptions of juiciness. However, it is plausible that the observed loss was associated with oil loss during cooking, given that the Mangalica produces fatty pork cuts, and such loss may not be reflective of water-holding capacity. These data generally support the hypothesis that RAC improves key pork quality attributes without impairing sensory characteristics.

Niche markets have steadily gained market share for food products during the last few decades as consumers have been willing to pay more for meat products that they perceive to provide a higher-quality eating experience [4]. However, such consumers also consider other aspects including the perceived safety and nutritional content of the product while the use of growth promotants could potentially be considered a negative practice [4]. Indeed, while the use of ractopamine hydrochloride is allowed in the U.S., Canada, and Mexico, its use is not permitted in several other global markets including those within the European Union (EU), Taiwan, China, and Russia. Such regulatory actions limit the markets that can be targeted by U.S. producers who utilize ractopamine while its use could potentially impact niche consumer choice within allowable markets. Despite such concerns, research has indicated that when used in compliance with established guidelines, pork from ractopamine-treated pigs is safe for consumers [4,20]. Indeed, recent literature suggests that consuming meat from ractopamine-treated animals might confer positive health benefits [44,45]. Furthermore, the use of clear and transparent labeling indicating whether pork products are derived from animals raised with ractopamine can, by an effective marketing strategy that drives consumer trust, help to overcome potential negative perceptions within niche markets [46]. Regardless, producers should carefully consider their markets and marketing strategies when choosing to adopt management strategies such as the use of growth promotants.

## 5. Conclusions

Given the growing interest of U.S. producers in adopting the Mangalica pig to target niche markets, there is great need to identify strategies that allow such producers to sustainably produce Mangalica pork while maintaining the carcass qualities that make the breed a viable option. This work aimed to address several limitations facing Mangalica producers in the U.S. market by testing the ability of RAC to improve growth performance without impairing pork quality attributes in this heritage breed. Feeding RAC significantly improved ADG, gain efficiency, LEA, and objective color scores without impairing marbling score or an objective measure of tenderness. These data indicate that Mangalica pigs are highly responsive to RAC and support the hypothesis that feeding RAC at an inclusion rate of 20 mg per kg for 21 days during the growing phase represents a viable strategy to improve the economic feasibility of utilizing this breed to target niche markets.

## Figures and Tables

**Table 1:** Composition of diet (as-fed basis).

Diet	
Ingredient, g/kg	
Corn	727.00
Soybean meal, 47.5% CP	107.00
Dried Distillers Grains	100.00
Dicalcium Phosphate	0.16
Limestone	11.51
Salt	4.00
Vitamin-trace mineral premix	0.45
Soybean oil	46.00
Calculated composition	
ME, mcal/kg	3.47
Crude protein, %	14.80
Fat, %	4.60
Ca, %	0.68
Available *p*, %	0.45

**Table 2:** Growth performance of Mangalica pigs fed 0 or 20 mg per kg RAC following 21 days ^1^.

Variable ^1^	Control	Ractopamine	*p*-Value
Initial body weight, kg	73.30 ± 3.20	72.20 ± 3.40	0.810
Final body weight, kg	84.30 ± 2.90	85.90 ± 3.00	0.720
Daily feed intake, kg	2.18 ± 0.10	2.23 ± 0.10	0.710
Average daily gain, kg	0.525 ± 0.039	0.652 ± 0.041	0.037
Gain efficiency ^2^	0.241 ± 0.015	0.292 ± 0.016	0.028

^1^ Values are group mean ± SEM, n = 14 per treatment; ^2^ Gain efficiency is expressed as kg gain/kg feed intake.

**Table 3:** Carcass parameters and primal cuts of Mangalica pigs fed 0 or 20 mg per kg RAC ^1^.

Variable ^1^	Control	Ractopamine	*p*-Value
Hot carcass weight, kg	75.60 ± 1.20	77.60 ± 1.20	0.220
Cold carcass weight, kg	74.00 ± 1.20	75.90 ± 1.20	0.250
Carcass length, cm	73.70 ± 1.60	71.20 ± 1.80	0.260
Ham, kg	15.13 ± 0.70	15.72 ± 0.80	0.210
Loin, kg	24.86 ± 1.30	25.54 ± 1.30	0.430
Shoulder, kg	14.41 ± 1.00	14.95 ± 1.10	0.450
Belly, kg	16.36 ± 0.80	16.54 ± 0.90	0.730

^1^ Values represent group mean ± SEM with n = 14 per treatment.

**Table 4:** Carcass composition of Mangalica pigs fed 0 or 20 mg per kg RAC ^1^.

Variable ^1^	Control	Ractopamine	*p*-Value
Loin eye area, cm ^2^	8.1 ^a^ ± 0.3	9.8 ^b^ ± 0.3	0.001
Muscle score ^2^	1.21 ± 0.12	1.44 ± 0.14	0.220
Average back fat, cm	5.30 ± 0.20	5.10 ± 0.20	0.410
Fat depth, cm			
1st rib	6.00 ± 0.20	5.70 ± 0.20	0.310
10th rib	5.70 ± 0.30	5.60 ± 0.30	0.900
Last rib	4.60 ± 0.20	4.50 ± 0.20	0.600
Last lumbar	5.20 ± 0.20	5.10 ± 0.20	0.610
Marbling score ^3^	2.23 ± 0.15	2.17 ± 0.16	0.770
Collagen (%)	3.00 ± 0.10	3.10 ± 0.10	0.150
Fat (%)	23.40 ± 1.50	21.20 ± 1.70	0.340
Protein (%)	22.30 ± 0.50	23.30 ± 0.60	0.210
Moisture (%)	63.10 ± 1.10	63.70 ± 1.20	0.680

^1^ Values represent group mean ± SEM with n = 14 per treatment while differing superscripts within a variable indicate differences between control and ractopamine groups when *p* < 0.05; ^2^ Muscle score: measured in ½ point increments with 1 = Thin and 3 = Thickest; ^3^ Marbling score: 1 to 2.4 = Devoid; 2.5 to 4 = Traces; 4 to 5 = Slight.

**Table 5:** Meat quality traits of Mangalica pigs fed 0 or 20 mg per kg RAC ^1^.

Variable ^1^	Control	Ractopamine	*p*-Value
Loin Ultimate Ph ^2^	5.74 ± 0.09	5.84 ± 0.10	0.470
L*, lightness	55.39 ± 1.28	51.92 ± 1.41	0.080
a*, redness	19.40 ± 0.31	18.94 ± 0.34	0.300
b*, yellowness	15.76 ^a^ ± 0.34	14.63 ^b^ ± 0.38	0.040
Cook Yield, %	20.40 ^a^ ± 0.60	18.10 ^b^ ± 0.70	0.020
WBSF, N ^3^	26.20 ± 1.20	24.30 ± 1.40	0.310

^1^ Values represent group mean ± SEM with n = 14 per treatment while differing superscripts within a variable indicate differences between control and ractopamine groups when *p* < 0.05; ^2^ Ultimate pH: measured 24 h post harvest on the chilled carcass; ^3^ WBSF: Warner–Bratzler shear force.

## Data Availability

Data are contained within the article.
